# Changes in capture availability due to infection can lead to detectable biases in population-level infectious disease parameters

**DOI:** 10.7717/peerj.16910

**Published:** 2024-02-29

**Authors:** Iris A. Holmes, Andrew M. Durso, Christopher R. Myers, Tory A. Hendry

**Affiliations:** 1 Department of Microbiology, Cornell University, Ithaca, NY, United States; 2 Cornell Institute of Host Microbe Interactions and Disease, Cornell University, Ithaca, NY, United States; 3 Department of Biological Sciences, Florida Gulf Coast University, Ft. Myers, FL, USA; 4 Center for Advanced Computing & Laboratory of Atomic and Solid State Physics, Cornell University, Ithaca, NY, United States

**Keywords:** Mark recapture, Parasitism, Capture bias, Individual based simulation

## Abstract

Correctly identifying the strength of selection that parasites impose on hosts is key to predicting epidemiological and evolutionary outcomes of host-parasite interactions. However, behavioral changes due to infection can alter the capture probability of infected hosts and thereby make selection difficult to estimate by standard sampling techniques. Mark-recapture approaches, which allow researchers to determine if some groups in a population are less likely to be captured than others, can be used to identify infection-driven capture biases. If a metric of interest directly compares infected and uninfected populations, calculated detection probabilities for both groups may be useful in identifying bias. Here, we use an individual-based simulation to test whether changes in capture rate due to infection can alter estimates of three key metrics: 1) reduction in the reproductive success of infected parents relative to uninfected parents, 2) the relative risk of infection for susceptible genotypes compared to resistant genotypes, and 3) changes in allele frequencies between generations. We explore the direction and underlying causes of the biases that emerge from these simulations. Finally, we argue that short series of mark-recapture sampling bouts, potentially implemented in under a week, can yield key data on detection bias due to infection while not adding a significantly higher burden to disease ecology studies.

## Introduction

Emerging infectious diseases, driven by climate change, introduced species, and other anthropogenic disturbances, are a conservation concern for many animal populations (Lafferty & Gerber, 2002; Smith, Acevedo-Whitehouse & Pedersen, 2009; Morand, 2020). Pathogens can impose strong fitness consequences on hosts, potentially reducing population growth and impacting long-term stability (Maslo & Fefferman, 2015; Iverson et al., 2016; Campbell et al., 2018). In addition, selection by parasites on hosts can lead to conservation-relevant evolutionary changes if parasite resistance is heritable (Prugnolle et al., 2005; Fumagalli et al., 2009; Kariuki & Williams, 2020). For example, a population that can quickly adapt to a novel disease may require less active management than a population that cannot (Spielman et al., 2004; Mikheyev et al., 2015). Conversely, strong selection toward infection-resistant genotypes may lead to reduced population-level genetic diversity (Jordan et al., 1998; Lenz et al., 2016). However, estimating the strength of selection or its correlates in wild populations is logistically challenging (Chao, 1989; McDonald & Amstrup, 2001; Grimm, Gruber & Henle, 2014).

A central challenge in connecting parasite infection to host fitness in natural populations is that infection can alter capture rates of hosts (Benton & Pritchard, 1990; McPherson et al., 2012; Garamszegi et al., 2015). Mark-recapture approaches are the state of the art in accounting for differences in capture likelihood between subgroups within a focal population. Robust design mark-recapture methods track the capture history, or patterns of detection and non-detection across sampling bouts, of individual animals over several consecutive bouts of sampling. For example, if an individual is sampled in bouts one and three, it can be assumed it was present during bout two but was not captured. The ratio of successful captures to misses of animals with known characteristics can then be used to calculate the capture rates for pre-identified subsections of the study population (Pollock et al., 1990; Nichols, 1992; Willson, Winne & Todd, 2011).

Parasites can impact host capture availability in a variety of ways. Parasites can reduce escape performance (Benton & Pritchard, 1990; Schall, 1990), causing a reduction of host activity or increase in risk aversion and thereby a reduction of capture rates (Bass & Weis, 1999; McPherson et al., 2012; Koprivnikar & Penalva, 2015). Conversely, the energetic demands of parasite infection could drive the host to greater foraging efforts, increasing availability for capture (Benton & Pritchard, 1990). Some parasites manipulate host behavior to increase risk of predation (Levri & Lively, 1996; Lagrue et al., 2007), which could increase capture rates of infected hosts. In addition, simple sampling error can impact estimates of key outcomes. This is particularly true when sampling from bounded distributions, when a parameter can vary freely in a specific range of values but not outside those values. These distributions are common in biology, when parameters are often constrained to positive values, for example the concentration of a protein (de Franciscis, Caravagna & d’Onofrio, 2014), the population size of an animal (Cai & Geritz, 2020), or the frequency of an allele in a population (Kimura, 1957). As such, identifying the ways that bounded distributions impact downstream analyses is of broad concern in biology, as well as specific concern in the context of our current work.

Here, we focus on several correlates of pathogen-driven selection that can be measured in wild host populations. When a biologically plausible resistance allele has been identified, quantifying changes in allele frequencies between generations can provide strong evidence of selection occurring (Westerdahl et al., 2004; Thrall et al., 2012). In addition, determining whether differing rates of infection are associated with different alleles or genotypes can provide evidence of selection (Langefors et al., 2001; Froeschke & Sommer, 2005; Dionne et al., 2009; Sin et al., 2014). Demonstrating differential reproductive success based on infection state is also critical, as some parasites do not impact lifetime reproductive success and so cannot drive selection (Schall, 1983; Gustafsson et al., 1994; Zylberberg et al., 2015). Calculated values of these correlates sampled from natural populations could be impacted by sampling error and bias.

Our study is motivated by the biology of one frequently studied family of vertebrate genes that can contribute to parasite resistance, the major histocompatibility complex (MHC). MHC proteins are responsible for recognizing pathogens and starting the adaptive immune response cascade (Kaufman, 2018). High MHC diversity can increase fitness by allowing an animal to mount immune responses to a broader variety of pathogens (Agudo et al., 2012; Radwan et al., 2012). However, in some systems a specific MHC allele will confer the strongest fitness benefit (Froeschke & Sommer, 2005; Wroblewski et al., 2015). Other gene families that are less studied than MHC, but may experience similar switches between directional and balancing selection due to parasite pressure, include the immunoglobulin A genes as well as scent and taste receptors, which play a role in recognizing pathogens in many tissues in the body (Sumiyama, Saitou & Ueda, 2002; Shi et al., 2003; Seixas et al., 2012; Carey & Lee, 2019; Harmon, Deng & Breslin, 2021).

We use a simulation approach to identify scenarios in which estimates of parasite-induced selection may lead to spurious conclusions due to sampling biases and error and discuss how these processes impact the values of our outcomes of interest. We establish a simulated population based loosely on the ecology of lizard-malaria systems. In such systems, parasite infection reduces host lifetime reproductive success but does not shorten host lifespan (Dunlap & Schall, 1995; Eisen, 2001), a common pattern for sublethal parasites (Dyrcz et al., 2005; Marzal et al., 2005; Hillegass, Waterman & Roth, 2010). We examine both heterozygote-advantage and resistance-allele advantage scenarios. The heterozygote-advantage simulations are an analog for the MHC-diversity advantage scenarios discussed above. The resistance-allele simulations model instances in which a single allele conveys protection against a pathogen. We quantify the impact of random subsampling and biased detection on our ability to estimate three outcomes of interest: 1) the fitness impact of infection, 2) the relative risk of infection of different host genotypes, and 3) changes in allele frequency from our parental generation to offspring generation.

## Materials and Methods

### Simulation framework

**Figure 1:**
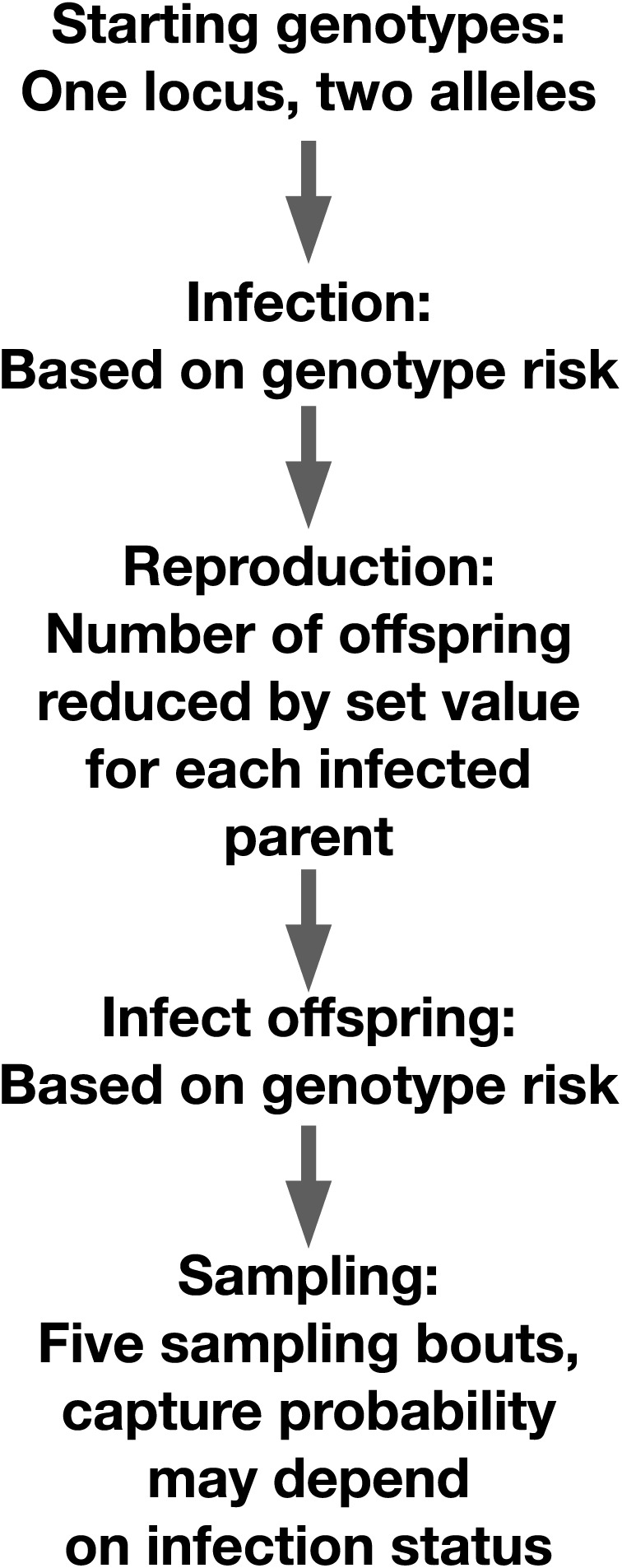
Schematic of our simulation setup. Organization of our individual-based simulation.

We simulated a diploid host population of 5,000 individuals with two alleles at a single locus in the R v4.4.1 scripting environment (R Core Team, 2021). Genotypes were generated using binomial random trials with equal probabilities of generating either allele (Fig. 1). Individuals were exposed to infection using a Bernoulli trial. The individual’s probability of infection in that trial was determined in part by their genotype. Each simulation had at least one genotype that conveyed resistance to a pathogen. In the ‘heterozygote’ runs, heterozygotes had lower infection risk, while both homozygous genotypes had higher infection risk. In the ‘resistance allele’ runs, carriers of a resistance allele, whether heterozygous or homozygous, had lower infection risk. For each run, we assigned a value between zero and one that described the degree to which genotype predicted infection risk. We varied this value for the simulations focused on allele frequency change across generations and per-genotype relative risk of infection. We selected this value to vary because it directly impacted the outcomes of interest, so perturbing it allowed us to test the reliability of our predictions across different scenarios.

To calculate the final value for an individual’s infection risk, we assigned a value for infection probability to the genotype. Across all runs, we used 0.8 for the high-risk genotype and 0.2 for the low-risk genotype. We multiplied the proportion by a “genotype prediction value” between zero and one that represented the degree to which genotype predicted infection risk as opposed to infections occurring at random. To represent the random component of infection risk, we drew a unique value for each individual from a normal distribution with mean of 0.5 and standard deviation of 0.2. We then multiplied that value by one minus the genotype prediction value and summed the two components. The final value was the product of individual genotype risk and the genotype prediction value added to the product of the inverse of the genotype prediction value and a random component. We truncated this final value between zero and one. We generated infections using a binomial random trial with the individual infection risk as the probability of success.

After infection, pairs of parents were randomly sampled from the population. For all pairs of parents, we assigned a number of offspring generated using the function ‘rpois’ in base R. The function requires a value ‘lambda,’ which describes both the expectation and variance of the function. The base lambda value for all simulations was 10. For each infected parent in a pair, we multiplied the lambda value by an infection penalty, which could vary across runs. The penalty took on a value from 0 to 1. For one infected parent, we would calculate the lambda value by taking half of the offspring (5), multiplying that value by the infection penalty, then adding the rounded value to the other half of the offspring. For two infected parents, the full lambda value (10) would be multiplied by the infection penalty. The parents’ genotypes were randomly subsampled to create gametes for offspring genotypes. Once offspring had been generated for 2,500 breeding pairs, the offspring pool was subjected to infection as described above and added to the full population. In our simulation, reproductive success was impacted directly by infection state but not by the parents’ genotypes. For our simulation runs focused on measuring reproductive success, we perturbed the parameter that controlled the expected proportion of offspring lost due to infection in the parents. We selected values from a uniform distribution between zero and one for this parameter.

We conducted five sampling events in which we randomly drew a set number of individuals, in this case 500, from the full population. To test the impact of sample size on our outcomes, we re-ran the simulations using sample sizes of 50, 100, and 1,000. Drawing small samples from a large population means that any single individual is unlikely to recaptured multiple times. However, mark-recapture statistics rely on individuals being sampled multiple times. To achieve this sampling structure, we used full population sizes of 500, 1,000, and 5,000 individuals for the 50, 100, and 1,000 sample size simulations, for a total of four separate population size/sample size combinations. We applied a Cormack-Joly-Seber model implemented in the R package ‘marked’ to these data to identify whether differences in capture rates between infected and uninfected individuals could be detected (Laake, Johnson & Conn, 2013). To generate capture histories for instances in which capture rates differed between infected and uninfected individuals, we first separated the infected and uninfected individuals. We found the number of infected individuals we expected to sample by multiplying the proportion of the population that was infected by our capture bias value for the run. We then selected individuals to be sampled using a uniform distribution implemented using the function ‘sample’ in base R. We applied the same approach to the non-infected individuals, sampling enough individuals to make up the final sample size. Finally, we performed control sampling on all individuals with identical capture values applied.

For recapture events, we did not differentiate between previously captured individuals and those that were not previously captured. We repeated the capture simulation steps until we reached our designated number of sampling events, in this case five. We then applied the CJS model to calculate capture probability for infected and uninfected individuals in these samples. We tracked genotype frequencies in the parents and offspring, and infection rates by genotypes in the pooled population. We assumed that the investigator is able to assign offspring to parents with no error, for example using parentage assignment analysis with neutral DNA markers (Wang, 2017, 2019). We describe potential real-world challenges with this assumption in our discussion.

We examined the effects of both random and biased sampling on three quantities of interest that are used in the disease ecology literature. First, we looked at the impact of infection on reproductive success, measured by the number of offspring detected from infected compared to uninfected parents. Second, we looked at the relative risk of infection in hosts with different genotypes. Relative risk is measured by dividing the proportion of infected individuals in the high-risk genotype by the proportion of infected individuals in the low-risk genotype. Relative risk values indicate the relative likelihood that the individuals in the focal class are infected compared to those outside that group. Finally, we calculated whether changes in allele frequency between parent and offspring generations due to differential reproductive success driven by infection could be detected with our sampling scheme. For each of our three metrics, we obtained values for the full simulated population (full), an unbiased subsample of the population (control sample), a subsample in which infected individuals were more likely to be captured (increased capture rate), and a subsample in which infected individuals were less likely to be captured (decreased capture rate).

We performed 200 complete runs of the model, including reproduction, infection, and sampling, for each of our outcomes of interest. Therefore, our three outcomes of interest are calculated from different sets of model runs. In each run, we performed control sampling in which all individuals were equally likely to be captured, as a comparison to the biased sampling outcomes. Our output files paired the biased and unbiased sampling results, so that the results can be directly compared. We randomly drew 200 values from two different uniform distributions for a parameter that described the proportional difference in capture rates between infected and uninfected hosts. One distribution was between 0.1 and 0.9 (reduced capture rate), and one between 1.1 and 1.9 (increased capture rate), while our control runs had no difference in capture rate between infected and uninfected hosts. We recorded the results of these simulations and uploaded the results, along with the code, on Zenodo (DOI:10.5281/zenodo.8067181). The values of the statistical tests in the results section are derived from these 200 recorded runs. For the sake of visual clarity, the figures are based on the first 50 runs in the outputs.

### Statistical tests

For each of our three outcomes of interest, we applied the same set of statistical tests to the values derived from the full population compared to the control samples, the increased capture rate samples, and the decreased capture rate samples. For the genotype relative risk and allele frequency change simulations, we performed separate analyses on the heterozygote and resistance allele runs. We used a slope test implemented in the R package ‘smatr’ to identify whether the regression of the increased rate, reduced rate, or control samples measured against the true values had a slope significantly different than one to one (Warton et al., 2012). The slope test returns an estimated slope of a regression with confidence intervals and a statistical test of the probability that the slope is equal to a given test slope. To test for greater variance in the increased and decreased capture rate samples relative to the values from the full population and control samples, we used a Fligner test implemented in the R package ‘stats’ (R Core Team, 2021).

Finally, we used two approaches to determine whether the Cormack-Joly-Seber mark-recapture model correctly identified runs with greater sampling bias imposed by the differences in capture rates. First, we used the CJS algorithm to calculate the capture probabilities for the infected and uninfected individuals in each run. We found the difference between the calculated capture probability values for the infected and uninfected groups. We then performed a t-test in base R between the control and increased rate differences and the control and decreased rate differences. This test showed whether the CJS capture rate values correctly identified altered capture availability between groups in our simulation runs.

Second, we found the residuals of a linear regression between the values for the parameter of interest from each of our three captured samples on our full-population values. We regressed the residuals against the absolute difference in the CJS model capture probability values for infected *vs* uninfected hosts. We report raw *p*-values, but we perform this type of comparison ten times in this paper, so a true significant *p*-value should be considered 0.005 with Bonferroni correction. A positive correlation between the difference in capture rate and the residuals for the parameters of interest would indicate that capture bias could impact the parameter. For our simulations focused on the reproductive success differences between infected and uninfected parents, we found that our sampling impacted the magnitude but not the proportion of the difference in reproductive success between parents in different infection categories. We found we could account for this sampling error by dividing the number of offspring sampled from all parents by the mean number of offspring from uninfected parents.

## Results

### Fitness impacts of infection

**Figure 2:**
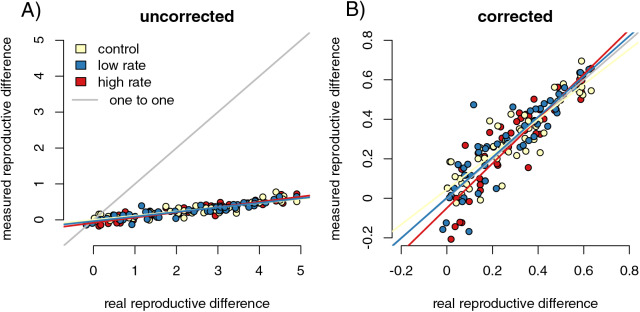
Sampling effects on the detectability of the impact of infection on reproductive success. Point colors represent different host reactions to parasite infection: 1) control samples in which infected and uninfected individuals have equal capture probabilities, 2) reduced capture rate samples in which parasitized individuals have a lower capture probability, and 3) increased capture rate samples in which parasitized individuals have a higher capture probability. Uncorrected sampling underestimates the impact of infection due to the uneven bounding of the parameter space (A). Correcting the measured values by dividing the differences in reproductive success by the mean number of offspring for uninfected parents reduces the sampling effects (B).

For each of the 200 runs of our simulation, we calculated the difference in mean number of offspring for infected and uninfected parents. We used slope tests to detect whether differences in capture rates between infected and uninfected hosts influenced the accuracy of the estimation of fitness reductions in infected parents. We first regressed raw values of the difference in offspring number from the control sample (equal capture rates between infected and uninfected individuals) relative to the values from the full population. We found that the sampled values dramatically underestimated the magnitude of the difference between reproductive success of the infected and uninfected parents (Fig. 2A, slope = 0.15 (0.139, 0.158), *p* = 0, F = 10,195.91, r = −0.99). When we divided the difference in offspring numbers in the control samples by the mean number of sampled offspring for uninfected parents (Fig. 2B), the value of the slope of the regression of the control samples against the full population values was much closer to one (slope = 1.16 (1.085, 1.230), *p* = 9.24 × 10^−6^, F = 20.73, r = 0.31). That is, proportional measures were not impacted by sampling but the calculated values of raw numbers of offspring were. The underestimation was likely due to the asymmetrical sampling space available. Parents with no offspring will always have their success ‘correctly’ detected by sampling, while the success of parents that do reproduce will be underestimated when some offspring are not captured.

We repeated the slope tests for the increased and decreased capture rate simulations. We found that slopes from corrected increased (slope = 0.998 (0.910, 1.094), F = 0.003, r = −0.004), and decreased (slope = 0.992 (0.909, 1.083), F = 0.032, r = −0.013) capture rate samples were not significantly different from one (increased *p* = 0.960; decreased *p* = 0.859). Since there is a stochastic component to infection for both parents and offspring, even susceptible parents with susceptible offspring are likely to have offspring in both infection categories. As a result, there are likely an adequate number of offspring in all infection categories to correctly detect relative reproductive success values even with biased sampling.

To test whether sampling resulted in a larger variance of outcome values relative to the corrected true population values, we performed Fligner tests comparing the corrected reduced rate, increased rate, and control samples to the true population values. Only the test of the control sample compared to the real population was significant (*p* = 0.050, med chi^2^ = 3.843, df = 1), while the increased and decreased rate samples did not have significantly higher variance than the real population (increased *p* = 0.103l, med chi^2^ = 2.653, df = 1; decreased *p* = 0.094, med chi^2^ = 2.804, df = 1). This is likely due to the altered sampling rates clustering outcomes together, thereby counteracting the variability caused by random sampling effects. Some of 50- and 100-sample size runs for both the slope test and the Fligner test were significant, likely due to greater impact of random sampling errors in smaller samples (Table S1). No Fligner test was significant for the 1,000 sample simulation.

We tested whether we could successfully differentiate the capture rates for infected and uninfected individuals in simulation runs in which capture availability differed for the two groups. This test ensured that lack of correlation between CJS capture values and our metrics of interest were due to characteristics of the metric, not a lack of differentiation between groups in the CJS values. T-tests successfully separated the differences in the capture rates of infected and uninfected individuals between the control and both varied capture rate runs (reduced rate: *p* < 2.2 × 10^−16^, t = 21.57, df = 265.53; increased rate: *p* < 2.2 × 10^−16^, t = −16.48, df = 373.57). We also determined whether we could detect a relationship between the difference in capture rates in a run as calculated by the CJS algorithm and the degree of error or bias in the reproductive success value in our sampled populations compared to the full population. We first found the residuals of a linear regression of reproductive success difference values from the captured samples on those from the full populations. These measured the degree to which the outcomes of a simulation run differed from the expected outcomes at a specific parameter value. Then, we found the absolute differences in the CJS capture probability values for the infected *vs* uninfected groups in each simulation run. We found the slope and R^2^ goodness of fit between the residuals and the capture probability differences. None of the three parameter values showed a significantly positive slope (increased *p* = 0.8189, F = 0.0525, df = 198; decreased *p* = 0.051, F = 3.858, df = 198; control *p* = 0.283, F = 1.158, df = 198), and all had small R^2^ values (increased R^2^ = −0.005; decreased R^2^ = −0.014; control R^2^ = −0.0007), indicating that CJS capture probability values cannot detect the sampling issues that cause spurious results when detecting reproductive success differences in real populations.

### Relative infection risks of host genotypes

**Figure 3:**
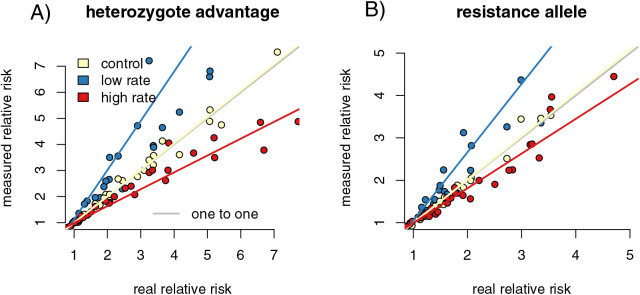
Differing capture rates impact estimates of relative infection risks of susceptible and resistant genotypes, in both the heterozygote advantage and resistance allele scenarios. Point colors represent different host reactions to parasite infection: 1) control samples in which infected and uninfected individuals have equal capture probabilities, 2) reduced rate samples in which parasitized individuals have a lower capture probability, and 3) increased rate samples in which parasitized individuals have a higher capture probability. In both heterozygote advantage (A) and resistance allele (B) scenarios, biased capture rates lead to some runs that dramatically under- or over-estimate true values. Relative risk is calculated by comparing numbers of individuals in four possible genotype-by-infection categories. The rarest infection by sampling category will be most vulnerable to sampling error, which could drive the spread we see in outcomes of the metric.

We calculated the relative risk of infection for individuals with the high-risk genotype compared to the resistant genotype for each of our capture rate scenarios. Regressing the relative risk measures from the control sample heterozygote runs against the full population resulted in a slope close to one (slope = 1.04 (1.013, 1.058), *p* = 0.002, F = 9.909, r = 0.216). The increased capture rate samples had a slope of 0.835 (0.815, 0.855), significantly lower than the control samples (*p* = 0, F = 225.094, f = −0.729). The reduced capture rate samples had a slope of 1.56 (1.493, 1.629), significantly higher than the control slope (*p* = 0, F = 428.372, r = 0.827) (Fig. 3A). The resistance allele runs had a similar pattern, with the slope of the control sample regression being 1.05 (1.035, 1.070) (*p* = 6.43 × 10^−9^, F = 36.836, r = 0.396) (Fig. 3B). The increased rate sampling significantly underestimated the size of the relative risk between different genotypes (slope = 0.755 (0.729, 0.782), *p* = 0, F = 258.889, r = −0.753), while the reduced rate samples significantly overestimated it (slope = 2.04 (1.936, 2.143), *p* = 0, F = 902.936, r = 0.906).

In the reduced capture rate simulations, infected individuals with the resistant genotype will be the rarest sampled category. The relative risk calculation divides the proportion of infected individuals with the high-risk genotype by the proportion in the low-risk genotype. Failing to accurately measure the number of infected individuals in the low-risk genotype will inflate the denominator of the relative risk calculation, driving up the final value. The rarest sampled category is the most vulnerable to random sampling error, particularly to under-sampling. This produces the triangle-shaped distribution visible in Fig. 3, in which some points in each increased and reduced rate samples fall near the control samples, but others deviate in the direction driven by under sampling the rare category. Similar logic applies to the simulations in which infected individuals are more likely to be captured.

According to the Fligner test, the variability in outcomes from the increased and decreased capture rate simulations in the heterozygosity runs was significantly greater than the variability of the full dataset (increased rate *p* = 0.037, med chi^2^ = 4.348, df = 1; decreased rate *p* = 0.006, med chi^2^ = 7.615, df = 1). The variance in the control samples were not significantly different from variance in the full dataset (*p* = 0.850, med chi^2^ = 0.36, df = 1). For the resistance allele runs, the pattern was similar, with differing rate samples being significantly different from the full dataset (increased *p* = 0.015, med chi^2^ = 5.861, df = 1; decreased *p* = 0.005, med chi^2^ = 7.911, df = 1), while the control sampling was not (*p* = 0.889, med chi^2^ = 0.020, df = 1).

**Figure 4:**
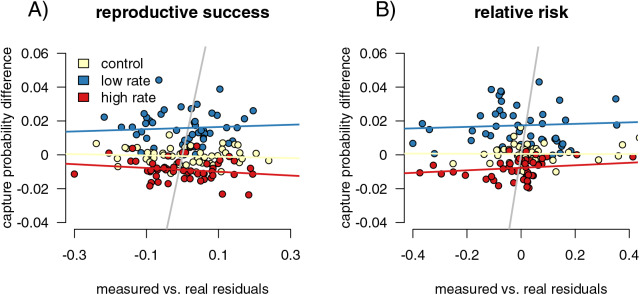
Mark-recapture approaches identify capture biases. We measured the ability of capture bias from CJS statistics to predict errors in our metrics of interest. First, we found the residuals of a regression of our metrics of interest (difference reproductive success for (A), and relative risk of infection for (B) calculated from the sampled individuals against the metric calculated from the full population. Higher residuals indicate higher bias. We then compared these residual values to metrics of capture bias from the CJS statistics. For measuring reproductive success differences, the residual values of the outcome of interest are not correlated with the success in detection of bias (A), while they are for detection of differences in genotype relative risk of infection (B).

T-tests again separated the differences between the capture rates of infected and uninfected individuals between control and varied capture rate in both simulations (increased het: *p* < 2.2 × 10^−16^, t = −16.41, df = 381.24; decreased het: *p* < 2.2 × 10^−16^, t = 19.863, df = 263.18, increased allele: *p* < 2.2 × 10^−16^, t = −19.11, df = 382.83, decreased allele: *p* < 2.2 × 10^−16^, F = 19.612, df = 254.71). Our linear models of residuals compared to capture probability values were significant for some parameter values (Fig. 4B; compare to the non-significant relationship between residuals and capture probabilities for the fitness impacts of infection in 4A). For the heterozygote simulations, the increased and decreased rate relationships were significant (increased: *p* = 0.004, F = 9.538, df = 198, R^2^ = 0.037; decreased: *p* = 2.6 × 10^−5^, F = 18.570, df = 198, R^2^ = 0.081), while the control runs were not (*p* = 0.084, F = 3.025, df = 198, R^2^ = 0.010). The resistance allele simulations followed a similar pattern, although the increased rate samples did not show a significant correlation (increased: *p* = 0.109, F = 2.597, df = 198, R^2^ = 0.008; decreased: *p* = 5.09 x 10^−16^, F = 78.22, df = 198, R^2^ = 0.280; control: *p* = 0.311, F = 1.034, df = 198, R^2^ = 0.0002). We take the CJS calculated estimates of capture probability for infected and uninfected groups and find that simulation runs with large differences in the CJS values for the two groups (runs with higher capture bias) also have higher deviations from expected value of our relative risk measure. Since we see this correlation, we hypothesize that high measured bias in a CJS study could flag instances in which calculated relative risk values should be treated with caution.

**Figure 5:**
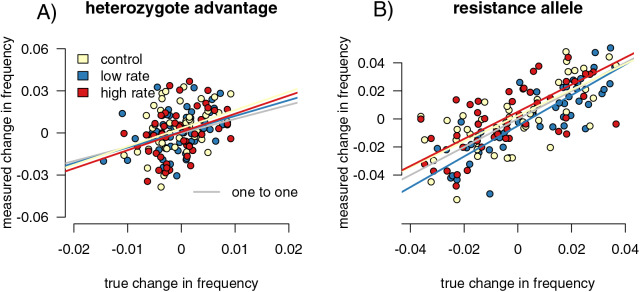
Sampling effects on measured changes in allele frequency between generations. Point colors represent different host reactions to parasite infection: 1) control samples in which infected and uninfected individuals have equal capture probabilities, 2) reduced rate samples in which parasitized individuals have a lower capture probability, and 3) increased rate samples in which parasitized individuals have a higher capture probability. In the heterozygote-advantage case (A), no linear relationship exists between measured and true allele frequency changes, and measured changes can be double true changes. In the resistance allele simulation (B), a relationship does exist, but the sampled population can still overestimate the true allele frequency change.

However, the relationships are relatively weak, though significant (Fig. 5B). In addition, the smaller sample size simulations (50 and 100 individuals sampled) did not show this significant correlation, indicating that higher sample sizes are necessary for this comparison to be useful.

### Allele frequency change detection

We found the difference in allele frequencies between our parental and offspring generations in each simulation. In the heterozygote-advantage simulations (Fig. 5A), allele frequency change calculated from the control samples showed a positive correlation with the true values (slope = 3.86 (3.402, 4.397), R^2^ = 0.891, *p* = 0, F = 765.911). The increased rate and decreased rate simulations behaved similarly (increased: *p* = 0, slope = 3.785 (3.313, 4.324), F = 674.354; R^2^ = 0.879; decreased: *p* = 0, slope = 4.024 (3.530, 4.589), F = 800.801, R^2^ = 0.895). In all simulations, sampling on average exaggerated allele frequency changes. In the resistance allele simulations (Fig. 5B), the slope was close to one, indicated that the sampled population directly reflected the direction and magnitude of allele frequency change in the true population (control = 1.291 (1.179, 1.414); increased rate = 1.309 (1.189, 1.441); decreased rate = 1.245 (1.130, 1.373)). Correlation coefficients were smaller compared to the heterozygote-advantage simulation (control R^2^ = 0.373; increased R^2^ = 0.415, decreased R^2^ = 0.368), and p-values were all significant (control *p* = 6.672 × 10^−8^, F = 31.499; increased *p* = 8.179 × 10^−8^, F = 31.040; decreased *p* = 1.355 × 10^−5^, F = 19.918). Differing capture rate samples had significantly more variance in allele frequency change than the full population in both heterozygote-advantage (increased *p* = 2.2 × 10^−16^, med chi^2^ = 110.55, df = 1; reduced *p* = 2.2 × 10^−16^, med chi^2^ = 139.4, df = 1) and risk-allele (increased *p* = 0.003, med chi^2^ = 8.859, df = 1; reduced *p* = 0.02, med chi^2^ = 5.653, df = 1) simulations. Control samples in both simulations also had significantly higher variance than the full population (*p* = 2.2 × 10^−16^ for both, het med chi^2^ =139.4, df = 1, allele med chi^2^ = 135.09, df = 1). With sample sizes of 1,000, the variance detected by the Fligner test was no longer significant (Table S1) for the resistance allele simulations but remained significant for the heterozygosity simulations.

As with all other comparisons, t-tests separated the differences between the capture rates of infected and uninfected individuals in all simulations (increased het: *p* < 2.2 × 10^−16^, t = −17.126, df = 385.1; decreased het: *p* < 2.2 × 10−16, t = 20.567, df = 253.97, increased allele: *p* < 2.2 × 10^−16^, t = −16.908, df = 378.71, decreased allele: *p* < 2.2 × 10^−16^, F = 19.257, df = 247.64). For the heterozygote runs, none of the regressions of CJS capture rates against residuals reached the threshold of significance (increased: *p* = 0.833, F = 0.044, df = 198, R^2^ = −0.005; decreased: *p* = 0.547, F = 0.364, df = 198, R^2^ = −0.003; control: *p* = 0.228, F = 1.463, df = 198, R^2^ = 0.002). The resistance allele simulations performed similarly (increased: *p* = 0.546, F = 0.365, df = 198, R^2^ = −0.003; decreased: *p* = 0.423, F = 0.646, df = 198, R^2^ = −0.002; control: *p* = 0.832, F = 0.045, df = 198, R^2^ = −0.004).

## Discussion

Infection-induced capture bias and sampling effects impacted the reliability of our three ecoimmunological metrics of interest in distinct ways. Differing capture rates between infected and uninfected individuals can inflate estimates of the relative infection risk associated with susceptible genotypes (Fig. 3), while sampling error can result in underestimating the impact of infection on reproductive success (Fig. 2). Sampling error can also inflate measured changes in allele frequencies between generations, leading to overestimation of the impact of selection due to parasite infection (Fig. 5). Sampling error is more severe with smaller sample sizes (Table S1). Mark-recapture statistics successfully identify simulation runs in which infected and uninfected individuals have different capture rates, and so can indicate problems for downstream analyses. For the relative risk of infection of different host genotypes, the difference in calculated CJS capture rates between the infected and uninfected hosts were positively correlated with outlier values in the metric in sampled relative to full populations (Fig. 5), indicating that they can be useful in identifying biased outcomes. The values were not correlated in the other two metrics. However, this level of discrimination is only possible at relatively high sample sizes, limiting its applicability with many taxa (Table S1).

Our simulated sampling underestimated the magnitude of the impact of parasitism on reproductive success (Fig. 2). We believe that this underestimate comes from the uneven bounding of the sampling distribution for this outcome (Kimura, 1957; de Franciscis, Caravagna & d’Onofrio, 2014; Cai & Geritz, 2020). An individual with no offspring will have their reproductive success “correctly detected” in every sample because no offspring are available for sampling. However, highly successful parents are most likely to have their reproductive success estimated to be far lower than their true value because they have more offspring in the population that can be missed. When an infection exerts selective pressure, uninfected parents should be the most reproductively fit. As a result, uninfected parents are the most likely to have their total success underestimated, which in turn dilutes estimates of selective pressure. Because the underestimates do not result from a difference in detection probability between infected and uninfected individuals, the difference in calculated capture rate values from the mark-recapture statistics are not helpful in identifying specific sampling events in which the issue is particularly acute. However, they can indicate instances in which caution should be applied to the interpretation of results. We find that adjusting for sampling effects by dividing the difference in reproductive success by the mean number of offspring captured from uninfected parents can nearly account for this source of error (Fig. 2B). Future work may be able to generalize this approach or develop different normalization approaches. Specifically, the bounded nature of the sampling error leads to nonlinear behavior by the outcome metrics. Here, we have both analyzed and corrected using a linear approach. Nonlinear methods may better capture this error distribution.

Our measures of the relative risk of infection associated with susceptible genotypes were inflated when infected individuals were less likely to be captured and biased down when infected hosts were more available for capture (Fig. 3). We believe this outcome resulted from a combination of the altered sampling rates and sampling error. When the values of rare classes, such as infected individuals with resistant genotypes, drive a metric of interest, the metric is highly susceptible to random sampling effects within the rare outcome class. Because this metric was directly impacted by capture availability, differences in capture values from mark-recapture statistics were able to identify more-impacted simulation runs.

Sampling error more than differences in capture rates impacted our ability to correctly detect the magnitude of allele frequency changes, particularly in our heterozygote-advantage simulation (Fig. 5). In our heterozygote-advantage simulations, true allele frequency changes in the population are less than half of the magnitude of the measured changes. In the resistance allele simulations, there is a relationship between measured and real allele frequency changes, and the amplitude of the allele frequency change can be more successfully detected from the samples.

Error in estimating parameters relevant to pathogen-driven selection on host populations could propagate into the estimation of a range of population-scale evolutionary scenarios. As with any organism, the pathogen population’s potential for adaptation is related to its size, which will in turn be impacted by the outcomes of host/pathogen coevolution. For example, the expected length of pathogen persistence in a host population is influenced by our focal parameters (Rand, 1995; Fleming-Davies et al., 2015). Overestimating the relative risk of infection to susceptible hosts might lead to the assumption that the host population will evolve toward resistance over short time frames (Lattorff et al., 2015; Vitale & Best, 2019; White et al., 2021). Pathogen population size might then be assumed to be lower than what it is in reality (Kao, 2006; Kerr et al., 2006), causing underestimation of the pathogen’s adaptive potential (Antolin, 2008; Gordo et al., 2009; Ailloud et al., 2019). Such incorrect inferences could impact short and long-term conservation planning for disease management (Frick et al., 2017). Epidemiological conclusions, particularly for poorly understood emerging disease, can also be impacted by incorrect inferences about local selection dynamics (Keeling & Gilligan, 2000; Ball et al., 2015; Britton & Scalia Tomba, 2019).

In addition to evolutionary potential, within-population behavior is key to understanding a pathogen’s metapopulation dynamics. Several parameters of metapopulation models, such as propagule pressure and the expected longevity of a host or pathogen population, are impacted by our outcomes of interest. Like other organisms, pathogens can experience local extinction events. Pathogen persistence at a landscape scale is therefore dependent on their ability to migrate between subpopulations of their hosts (Soubeyrand et al., 2009). Even if the pathogen will eventually go locally extinct, a longer than expected persistence will provide more opportunities to colonize naive host subpopulations and persist at the landscape scale (Laine, 2004). Metapopulation models are used to predict the spread and impact of emerging infectious diseases (Keeling & Gilligan, 2000; Ball et al., 2015; Britton & Scalia Tomba, 2019), predict the evolutionary trajectory of virulence (Thrall & Burdon, 2003), and determine the likelihood that some host subpopulations will remain uninfected (Dijk, Ehrlén & Tack, 2022).

Field studies have several sources of uncertainty that are not modeled here. First, false negative tests for pathogen presence can occur. Using mark-recapture techniques, the probability of non-detection of infection can be modeled jointly with imperfect detection of hosts (Jennelle et al., 2007; Conn & Cooch, 2009; Cooch et al., 2012; Tersago et al., 2012). Second, errors in inference of parent-offspring relationships can impact estimates of fitness. As with pathogen detection, uncertainty in genealogical reconstruction using DNA markers can be accounted for in empirical systems (Zhu, Fung & Guo, 2007; Lacy, 2012; Wang, 2017, 2019). Finally, spatial or other environmental heterogeneity could impact recapture rates. For example, if infection correlates with occupancy a specific habitat type that alters capture availability, biased sampling could occur even if infection doesn’t directly alter host behavior (Johnson et al., 2009; Hernández-Olascoaga, González-Solís & Aznar, 2022). In our simulation, every individual is equally likely to be infected. Real pathogens tend to move through populations through contact between individuals or through vectors (VanderWaal & Ezenwa, 2016; Mousa et al., 2021). These complexities could also result in non-random spatial patterns of infection, potentially introducing further bias in downstream analyses. Further, some of the effects that we note depend on sample size. As sample sizes get larger, approaching 500 or 1,000 individuals, samples better approximate the variability and slope of the full populations. Conversely, our ability to use mark-recapture statistics to detect events that are strongly impacted by sampling bias is only reliable at larger sample sizes. These sample sizes may be impractical for some study taxa. While our simulations represent a best-case scenario for information about individual hosts, they describe patterns of capture biases that can occur or even be inflated in work on natural populations.

## Conclusions

Identifying the possibility of parameter estimation errors due to differences in capture rate and sampling error is a key concern in expanding landscape-scale host-pathogen evolution studies to a broad range of species. Mark-recapture techniques provide a key detection probability metric that quantifies the likelihoods of encountering individuals with different biological traits. Many mark-recapture studies are intended to detect survival probability of animals over relatively long time scales. These studies are time and resource intensive, because they require a large enough sample size of individuals to ensure that some can be recaptured throughout the study. However, we demonstrate that a single, week-long, bout of robust-design sampling has considerable value in identifying detection bias between infected and uninfected individuals. Implementing this approach in the field could increase the accuracy of disease ecological sampling across many taxa.

## Supplemental Information

10.7717/peerj.16910/supp-1 Supplemental Information 1 Results of varying sample sizes on statistical outcomes. We show the impact of four sample sizes on the statistical outcomes discussed in our paper

## Additional Information and Declarations

### Data Availability

The following information was supplied regarding data availability: The code and example simulation output files are available at Zenodo: Holmes, Iris A, Durso, Andrew M, Myers, Christopher R, & Hendry, Tory A. (2022). Changes in capture availability due to infection can lead to correctable biases in population-level infectious disease parameters. https://doi.org/10.5281/zenodo.8067181.
